# FamilyGuard: A Security Architecture for Anomaly Detection in Home Networks

**DOI:** 10.3390/s22082895

**Published:** 2022-04-09

**Authors:** Pedro H. A. D. de Melo, Rodrigo Sanches Miani, Pedro Frosi Rosa

**Affiliations:** School of Computer Science, Federal University of Uberlândia (UFU), Uberlândia 38400-902, Brazil; miani@ufu.br (R.S.M.); pfrosi@ufu.br (P.F.R.)

**Keywords:** machine learning, anomaly detection, network security, smart home, Internet of things (IoT)

## Abstract

The residential environment is constantly evolving technologically. With this evolution, sensors have become intelligent interconnecting home appliances, personal computers, and mobile devices. Despite the benefits of this interaction, these devices are also prone to security threats and vulnerabilities. Ensuring the security of smart homes is challenging due to the heterogeneity of applications and protocols involved in this environment. This work proposes the FamilyGuard architecture to add a new layer of security and simplify management of the home environment by detecting network traffic anomalies. Experiments are carried out to validate the main components of the architecture. An anomaly detection module is also developed by using machine learning through one-class classifiers based on the network flow. The results show that the proposed solution can offer smart home users additional and personalized security features using low-cost devices.

## 1. Introduction

The accelerated growth of applications and devices for the Internet of things (IoT) means excellent amenities for people in diverse areas, such as smart homes, industrial automation, healthcare, electricity control, cities, and smart grids [1]. However, deploying IoT applications in different scenarios can also introduce security threats. An example is the Mirai botnet, first identified in August 2016 by a security research group called MalwareMustDie. Mirai scours the Web for smart home devices that have default usernames and passwords and then enlists the devices in attacks that hurl junk traffic at an online target [2].

IoT security is also affected by software and hardware constraints on such devices. For example, implementing encryption and authentication mechanisms on these devices can be challenging [3]. Another problem is that IoT applications might rely on users’ personal information to provide services. However, collecting, transferring, and using this information increases the risk of damaging users’ privacy [4,5]. Thus, one of the most prominent research challenges for the networking and security community is providing a cost-effective balance between the use of sensitive data and privacy [6].

There are several types of non-standard IoT devices and protocols in smart home scenarios whose lack of standardization can pose security risks to this environment. For example, the owner of cameras, TVs, and smart sensors must manage the security updates individually, even if they do not have the time or the knowledge to deal with this situation. Moreover, without proper threat monitoring, infected mobiles devices might compromise the smart home environment as they ingress into the home network.

Several works propose using SDN to create a safer environment for smart homes [7,8,9]. When dealing with security solutions for smart homes, we should consider several challenges when deploying the solution in the environment: heterogeneity of devices and protocols and resource constraints such as small memory, low power consumption, and low computing power. Based on these security challenges, several researchers pursue solutions that include privacy-based, risk-based, and role-based approaches [10,11,12].

The scientific community offers advances in the security of smart home networks [7,8,9,10,11,12]. However, most solutions do not show how security mechanisms could be deployed to operate in the residential environment. Therefore, there still exist research gaps in security requirements for smart home networks consisting of device authentication, network monitoring, secure session key management, physical protection, information security, and user authentication [13,14]. Another limitation identified in the state of the art is about solutions that detect anomalies. Usually, they do not present the complete structure of the solution; in other words, it is unclear how network traffic would be captured and classified.

Therefore, this work presents FamilyGuard, an architecture that provides a way to define and deploy mechanisms that meet the security requirements of a smart home environment. FamilyGuard uses the SDN paradigm to analyze and manage home area network (HAN) flows, providing flexibility when dealing with security issues, such as traffic monitoring to identify and mitigate threats. The architecture uses machine learning (ML) algorithms to identify anomalous behavior based on network traffic to provide additional protection to residential environments in terms of information security. Our idea is that the proposed architecture would help transform the static residential environment into a dynamic one. In other words, FamilyGuard will provide means to deal with changes in the environment and respond to threats.

Our contributions can be summarized as follows: (i) we design the building blocks of a machine-learning-based security architecture for smart homes, (ii) we propose and implement the components responsible for monitoring the traffic of a smart home network, (iii) we evaluate the feasibility of using one-class algorithms to identify smart home anomalies, and (iv) we evaluate some of the architecture capabilities using an empirical strategy based on a combination of public datasets and real-world equipment.

The remainder of this study is organized as follows. Section 2 provides a background on smart home networks, Internet of things, SDN, and IoT security issues. Section 3 presents a systematic literature review about security architectures for smart home environments. Section 4 shows the proposed architecture. Section 5 provides a set of experiments and exploratory investigations that validates some of the FamilyGuard functionalities. Section 6 discusses limitations and threats to the validity of our study. Finally, Section 7 presents the concluding remarks and future work.

## 2. Background

### 2.1. Smart Home Concepts

A smart home can be defined based on two perspectives. The first perspective considers a house equipped with Information and Communication Technology (ICT) and with connected devices that can be remotely monitored and controlled to meet the needs of residents. In the second perspective, smart homes and other related buildings are seen as elements of flexibly and interactively connected energy systems on a broader scale. These two perspectives are useful for the families themselves and the electrical system as a whole [15].

In the home environment, “intelligence” can integrate electrical devices and services (e.g., heating, lighting, security, photovoltaic generation, electric vehicle charging) that the occupants or other agents remotely control. In addition, sensors and processors can also obtain and apply knowledge about a house, operating independently of direct human action. Together, intelligent or smart household electrical devices have the potential to help manage the network and promote system efficiency, helping to reduce peak demand and match demand with supply in real time. This assists the integration of more distributed renewable generation into electrical systems [16].

There are questions about what technologies are needed at what times to qualify a home as smart. Smart TVs or smartphones, for example, can be classified as smart devices since they allow communication between the house and the outside world. In this study, we consider that a high level of device connectivity inside and outside the residential environment, together with the reliance on this connectivity for daily activities, is relevant to whether a home can be named “smart”. The definition presented here is associated with physical and operational factors and assumes that the functionalities are beyond the typical limits of a traditional house [17].

A smart home is a communication network that connects sensors, appliances, controls, and other devices to enable remote monitoring and control by occupants and others to provide frequent services to residents and the electrical system. Therefore, we can say that this concept is closely linked to the term Internet of things (IoT) because smart homes provide facilities for the occupants of the residential environment and interconnect objects and sensors.

### 2.2. Internet of Things (IoT)

The term Internet of things (IoT) refers to the network interconnection of objects (sensors, actuators, devices, etc.), which allows sharing of data and information to accomplish some task [18]. The term was also defined in the Recommendation ITU-T Y.2060 (June 2012) as “a global infrastructure for the information society, enabling advanced services by interconnecting (physical and virtual) things based on existing and evolving interoperable information and communication technologies” [19].

The advancement of IoT applications and devices is providing progress in many areas, each of them with different requirements and goals [1]. The smart home, for example, aims to provide greater convenience for people, the smart grid seeks to obtain higher efficiency, reliability, and sustainability in energy systems, and smart-agro offers solutions to improve productivity in agriculture.

As the objectives of these areas are entirely different, the security mechanisms employed in these environments need to be adapted to their needs. This work seeks to contribute to the domain of smart homes where there are several challenges, such as interoperability, context-aware middleware, energy-aware/efficient consumption, and security [20].

The FamilyGuard architecture proposed in Section 4 intends to find solutions to the security challenges of the smart home environment. Since the devices are interconnected using different network standards [21] (Zwave, Insteon, Bluetooth, Zigbee, Ethernet, Wifi), they can be targeted by several types of attackers, from cyber terrorists to script kiddies. Furthermore, smart homes are usually operated by non-expert users in the security of information [22]. Therefore, it is important that the solution involves a high level of automation at the lowest possible cost.

### 2.3. IoT Security Issues

Smart home environments are full of personal information handled by devices, protocols, and services to provide user convenience. Due to the sensitivity of private information, security requirements become more and more necessary in such a scenario. However, managing security controls in these heterogeneous environments is a significant challenge. According to Bugeja et al. [23], security issues can be divided into three different layers of the generic IoT architecture:

- Device Issues: devices in IoT scenarios have performance constraints such as CPUs with low clock rates, low memory, and low throughput. These hardware limitations make it difficult to implement security mechanisms, such as encryption, which are computationally consuming. Many devices do not have a management interface, making it difficult to create security mechanisms such as authentication. For this reason, users need to trust websites or smartphones to manage their devices and information. Another critical issue is that objects in a smart home are physically accessible and may be subject to physical attacks such as tampering by a visitor in the home or even by the household to reduce the charge for some service that relies on smart meters [23].
- Communication Issues: to interconnect many different devices in a smart home, multiple bridges, hubs, or gateways and many communication protocols are required, which makes it difficult to implement adequate security mechanisms. The smart home environment is highly dynamic, where a device can join or leave the network at any time, reinforcing the idea of developing resilient security mechanisms that can handle asset management. A large number of existing protocols and the restricted capabilities of each device make traditional security mechanisms unsuitable for the smart home environment [21].
- Service Issues: to reduce the number of vulnerabilities, patch fixes need to be installed periodically. However, performing this process on all devices could be problematic since the firmware of these devices and protocols may not support these security updates dynamically [23].

Based on these questions, we can conclude that implementing traditional security mechanisms to tackle problems in smart homes may not be the best strategy. Therefore, it is necessary to explore the use of alternative approaches. One of them is using a Software Defined Networking (SDN) that, when applied to the IoT context, will change the network from a static to an adaptive or programmable state, which is a necessary feature in a heterogeneous environment.

Several areas in IoT are gaining benefits with the SDN paradigm, for example, smart cities [24], smart grid [25], smart homes [26], among others. Kalkan and Zeadally [5] conclude that IoT challenges such as security, scalability, and heterogeneity can be solved through the dynamism and flexibility of SDN. However, integrating SDN and IoT environments will open up new security risks, such as attackers granting themselves unsupervised access to SDN elements and exploiting either weak or nonexistent access control mechanisms. Another example is the creation of exploits for vulnerable network components for the purpose of installing them on rogue devices or binding them to remote connections to the network. Researchers must strive to mitigate these threats in the future [27].

### 2.4. Software Defined Networking (SDN)

With SDN [28], networks became programmable, making it possible to virtualize network functions and manage services and applications through logically centralized platforms. SDN introduces control plane and data plane separation. One of the main goals of this separation is creating an agile and flexible network that is capable of handling rapid changes, supporting some requirements of IoT environments that traditional networks may not be able to provide [5].

The SDN controller is responsible for managing the entire network, while switches are responsible for operating the data plane based on the settings specified by the controller [29]. The Southbound API is offered by the OpenFlow protocol, and its assignment is to enable communication between the SDN controller (control plane) and network switches (data plane), thus allowing the controller to define the network flows and the API Northbound interface between the controller and higher layer applications or programs.

The SDN is materialized through the OpenFlow protocol [30] and has a set of specifications maintained by the Open Networking Foundation (ONF) [31].

Security solutions involving SDN can be categorized into two types: (i) security solutions for SDN, which aim to improve the security of protocols and controllers (control plane); and (ii) SDN security solutions that seek to improve network security using SDN (data plane) features [32]. Examples of security solutions for SDN are FortNOX [33], CloudWatcher, FRESCO [34], FlowGuard [35], Avant-guard [36], among others. Such solutions are not detailed because they are outside the scope of this work. Solutions that use SDN to provide new security features are detailed in Section 3.

We emphasize that our solution is easy to implement and that all its components are operable with low-cost devices; we evaluate a class of algorithms not explored by the related works and build a dataset that can represent the traffic of the residential environment.

## 3. Related Work

Several research projects worldwide seek to propose solutions to improve the security of residential environments. We conduct a systematic literature review to identify the main works and also the research gaps. First, keywords, such as smart home, security mechanisms, anomaly detection, etc., widely used in journal and conference articles, are adopted as input to search engines such as Google Scholar, IEEE Xplore, and ACM Digital Library. We select the following criteria for selecting articles: the number of citations, the journal’s impact factor, and the conferences’ maturity. Finally, the selected studies are organized according to the following characteristics: year of publication, placement strategy, validation strategy, possible threats, security goals compromised, countermeasures, techniques employed for developing countermeasures, and the existence of SDN resources. Later in this section, we describe each of these items together with a table that summarizes our systematic literature process. Next, we will discuss the selected studies.

Abu-Tair et al. [37] describe an architecture in its initial conception. The work evaluates cryptographic algorithms, which IoT devices can use before connecting them to the smart home architecture. This is an embryonic proposal that aims to create a prototype to test numerous applications in order to seek optimal performance and security for the devices.

Gordon et al. [38] use Field Programmable Gate Arrays (FPGA) to perform parallel processing. The proposed parallelization and implementation of the K-Nearest Neighbors (KNN) machine learning algorithm in FPGA are achieved using Vivado Design Suite from Xilinx and High-Level Synthesis (HLS). The proposed solution presents a better performance in FPGA when compared to four alternative KNN instances.

Ammi et al. [10] suggest a new Blockchain-based solution for smart home systems, using a combined hyperledger array and a hyperledger composer. This solution explains commonly used permissioned blockchain security limitations with permissions. The architecture contains four layers: cloud storage, Hyperledger Fabric, Hyperledger Composer, and a smart home layer. The architecture is implemented and tested to improve smart homes’ integrity, confidentiality, availability, authorization, and privacy.

Mascarenhas et al. [11] aim to reduce attacks on a smart home network and thus create a patrolling system for this epitome of urbanization. The proposed design consists of individual device proxies. The central hub pulls application layer data from every IoT device from every proxy device on the network. The collected data are used as the input to train the central hub to identify intrusions into the smart home network. The architecture uses XGBoost to categorize and detect anomalies. In addition, it implements a rule-oriented access mechanism to validate authorization and access controls.

Ameer et al. [12] show an access control model for IoT in the smart home. The authors provide a formal definition for extended generalized role-based access control (EGRBAC) and show its capabilities with a use case. EGRBAC is the initiative developing a comprehensive family of IoT smart home access control models. A proof-of-concept demonstration using AWS-IoT Greengrass is discussed in the appendix.

Augusto-Gonzalez et al. [7] present an architecture embedded in a smart device, adapted for home networks, and designed to be vendor independent. The conceptual design of the architecture involves advanced data flow analysis, which is able to classify user and device profiles; it can also be used for automated real-time risk assessment. Comparisons and matches with secure data flow patterns are made using a self-learning approach. Data analysis and visualization techniques are implemented to ensure greater user awareness and understanding of security status, potential threats, risks, associated impacts, and mitigation guidelines. The defined validation strategy is based on a triple view that combines laboratory tests, testbeds, and pilot tests in a real environment.

Sharma et al. [8] introduce an architecture that uses the SDN paradigm within the residential environment. Three case studies are used to demonstrate that the architecture can provide additional protection to residential environments. The first one is related to user privacy, the second is the use of the architecture to prevent DoS/DDoS attacks, and the third one suggests that the architecture can resolve security breach issues in digital voice assistants. The overhead and performance of SHSec are evaluated using packet loss rate, flow configuration time, and controller response time.

Alves et al. [9] present a service by using SDN to perform autonomous management of wired and wireless home networks. According to the authors, an ISP can use the solution to reduce customer network downtime. With this control, ISPs have a more comprehensive range of operations to troubleshoot remotely and therefore they reduce the amount of onsite maintenance. A prototype is evaluated, simulating common failures in home networks. As a result, the architecture increased throughput and reduced network delay.

Hafeez et al. [39] provide a service-oriented security solution using cloud computing and SDN to improve network monitoring, security, and management. Installed residential devices act as a sensor for the Cloud Security Service (CSS), which collects network-level statistics that can be used to perform analysis to detect botnets, malware, and other insights into the network. The cloud manager is the central component of the architecture, and its responsibility is to manage client requests, resources, and traffic analysis tasks. The authors also discuss how to evaluate the prototype in different scenarios such as latency, collaborative threat detection, privacy, efficiency, scalability, and bandwidth.

Demetriou et al. [40] present a solution partially inspired by the SDN paradigm, designed with the idea of being easily deployed in HAN. The architecture features distributed security control that includes a controller in a home router for policy enforcement and a monitor on the user’s phone to collect runtime status and make access decisions. To avoid changing the mobile operating system, the monitor is in the form of a user-space app. It detects the application communicating with the network and its compliance with security policies and sends the access permission to the router controller over a secure control channel. The router uses this information to enforce policies, being that only traffic with monitor permission can access IoT devices.

Stewart et al. [41] introduce the architecture CommunityGuard (CG) with two main components. The first one is the guardian node, responsible for observing all the traffic on the home network. The second is the community outpost, a cloud server that interfaces with guardian nodes and performs traffic monitoring. When it identifies a security issue, it will deploy defenses as needed. An initial prototype is created using the Snort IPS system to manage its configuration automatically. Together, these components provide collaborative security monitoring.

Table 1 summarizes the results of our systematic literature review process. Next, we detail the characteristics of our taxonomy.

- Name: name of the project, solution, architecture, or framework.
- Ref: reference of the analyzed work.
- Year: the year of publication of the work.
- Placement Strategy: the solutions are monitoring traffic and managing the network to improve security in the residential environment. It is essential to assess where these data are being processed and where they are being managed. If this processing/management occurs inside the house, it is considered the edge computing strategy; when the processing occurs outside the residential environment, we consider it the cloud computing strategy. The solutions can use both strategies that keep the analysis inside the home, but help can be sought in a cloud to perform the functions.
- Validation Strategy: knowing the form of architecture validation is essential for us to analyze how the solution is explored. Hypothetical validation has a distant relationship to a real environment, empirical validation uses information from operational configurations, experiences, and observations, experimental validation seeks to reproduce the scenario where the solution is being applied, and theoretical validation uses theoretical arguments to support the results.
- Possible Threats: threats that the solutions aim to solve.
- Security Goals Compromised: what can be compromised by threats.
- Countermeasures: identify the main contribution created by the presented solutions.
- Techniques/Tools: techniques/tools used to create countermeasures.
- SDN: identifies whether the selected reference adopted SDN resources. The • symbol means that the architecture supports SDN while the ∘ symbol indicates no SDN support.

Our solution has benefits concerning the works analyzed in Section 3. The structure of the FamilyGuard architecture is robust and flexible to meet the heterogeneity of residential environments. Its design allows SPPs to provide customized templates for each environment according to customer demand. Another important factor in our proposal is that FamilyGuard considers all devices present in residential environments, not just IoT devices like most architectures analyzed. The AI Workflow Orchestrator (AIWO) can configure and manage multiple workflows to meet the HAN. When it detects a threat, the Security Orchestrator (SECOR) runs to mitigate the problem.

## 4. FamilyGuard

FamilyGuard is a security architecture designed for smart homes equipped with different types of computing devices. The main goal of FamilyGuard is to anticipate and respond to the needs of residents, working to promote their comfort, convenience, safety, and entertainment. According to [42], home environments lack reliable security solutions since households occasionally only have antivirus software installed on computers and rarely have perimeter defenses installed on their networks such as an intrusion detection system (IDS) or a firewall. Besides, our systematic literature survey presented in Section 3 showed that proposed solutions only focus on IoT devices and ignore the other communication devices present in the HAN. Our solution serves the entire residential environment, detecting threats in IoT and mobile and traditional devices, such as computers and laptops.

**Figure 1:**
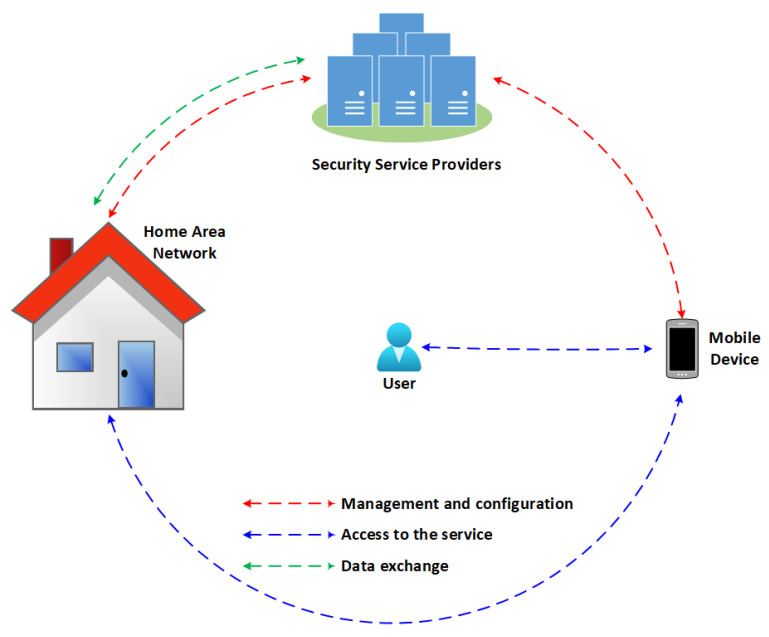
High level architecture. The user can access some of the security services provided by SSPs using mobile devices.

Figure 1 presents a high-level view of the architecture. Security service providers (SSPs) are responsible for providing management and configuration features that help protect existing information on a home area network (HAN), such as machine learning (ML) models to identify anomalies in network behavior. The user can access the services provided by SSPs through an application on their mobile device and hire a Security-as-a-Service (SECaaS) that meets their needs. HANs can send information and alerts to SPPs if they identify anomalous situations in the network. In this way, by managing multiple households, an SPP can work collaboratively to detect threats.

**Figure 2:**
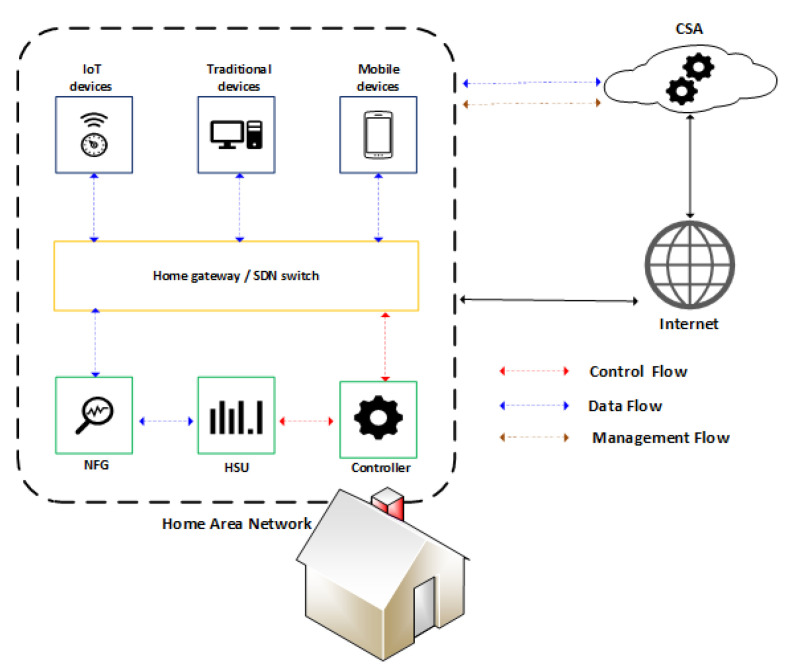
Communication between the architecture components.

Each family living in a smart home environment has specific needs. Therefore, besides dealing with the heterogeneity of devices, it is necessary to address the specificities of each smart home environment. We will tackle this issue by analyzing the inbound and outbound network traffic. With this in mind, we propose the following essential components of FamilyGuard: Home Surveillance Unit (HSU), controller, network flow generator (NFG), and Central Security Assistant (CSA), which are described respectively in the Section 4.1, Section 4.2, Section 4.3 and Section 4.4. Figure 2 shows each of the components in the environment and how they communicate.

The NFG receives network packets transmitted by the home gateway and generates network flows analyzed by the HSU. Upon identifying any anomalous behavior, the controller receives a notification, and the device involved in the anomalous behavior can be blocked to mitigate potential threats. The rules that determine the actions to be taken when identifying a threat depend on the HSU configuration; it may issue only a notification or completely block device communication.

### 4.1. Home Surveillance Unit (HSU)

**Figure 3:**
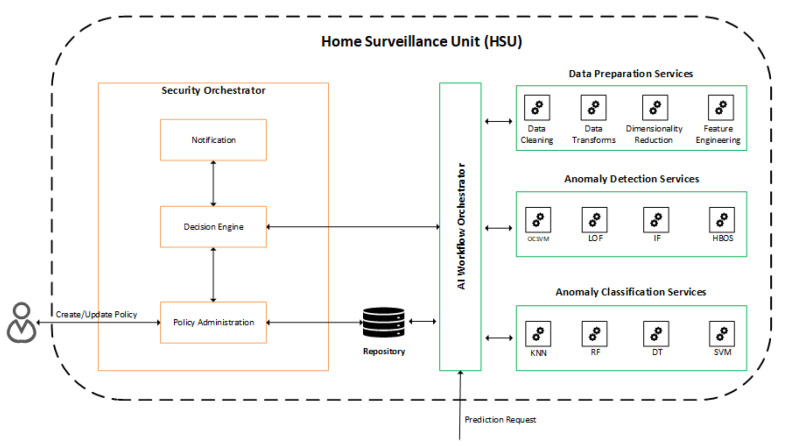
Communication between the services present in the HSU.

The HSU is responsible for receiving flows from the network, performing traffic analysis tasks, and managing the network through the controller interface. The HSU uses a workflow to handle incoming network traffic flows and perform analysis. A workflow can contain multiple artificial intelligence models to handle flows and perform analysis sequentially. For example, the HSU receives a flow and forwards it to Workflow A, which initially prepares the flow by removing unnecessary characteristics; another model identifies the traffic type of the flow and forwards it to the last model to verify if the flow has anomalous behavior. The structure HSU has two main components: the Security Orchestrator and the AI Workflow Orchestrator, shown in Figure 3 and described below:

- Security Orchestrator (SECOR): is responsible for managing, configuring, and providing notification information related to security policies applied in HAN. Upon receiving the result of a prediction from the AI Workflow Orchestrator, the decision engine checks the applicable security policies to defeat or mitigate the detected threat and then notifies the controller so that the change in the flow table is performed to block or limit access to the device on which the threat was detected. The user can also change the security policies, being able to choose to either block the infected device immediately or be notified and make the blocking decision later.
- AI Workflow Orchestrator (AIWO): in charge of receiving prediction requests and generating results for the Security Orchestrator, being also responsible for managing and administering services available for predictions, categorized as Data Preparation Services, Anomaly Detection Services, and Services of Anomaly Classification.

**Figure 4:**
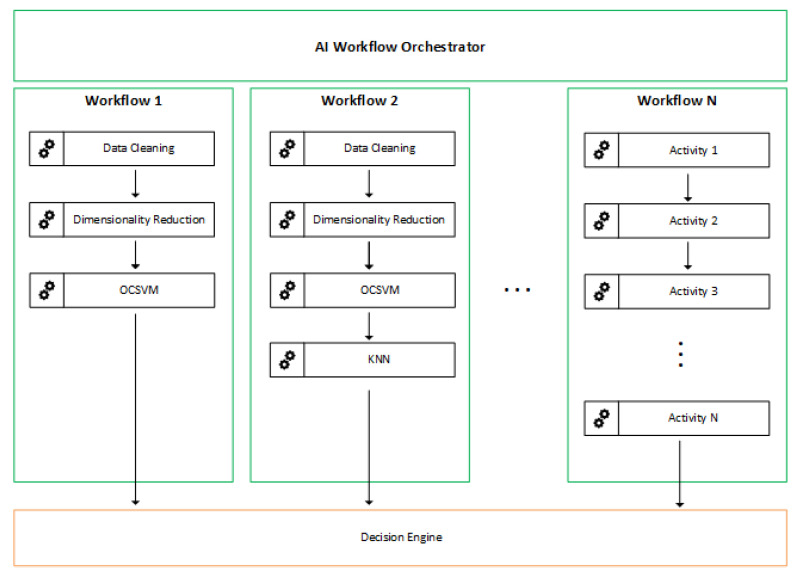
Workflow Orchestrator is responsible for managing several workflows.

Figure 4 exemplifies the structure of the AIWO, which allows for the definition of multiple workflows using a set of available models. SSPs and users are set free to create and define workflows to meet specific HAN needs with this structure. For example, we can have workflows that identify anomalies in specific scenarios, such as in IoT devices, and ignore traditional home devices, such as notebooks and personal computers.

It is essential to highlight that we can have several workflows in operation; however, a prediction request does not have to go through all workflows. AIWO allows a configuration in which flows from certain devices are routed to specific workflows. In this way, we can have models that identify anomalies at a lower level of granularity.

### 4.2. Controller

The SDN controller is installed in a computing device responsible for receiving the HSU instructions and relaying them to the home switch (HS), where all devices in the home must be connected using wired or wireless connections. The approach used in this work was to deploy the HSU and the controller on the same device. The main advantage of running the HSU with the controller was maintaining a simple deployment scenario in a smart home, avoiding using another device to execute the HSU instructions. Performance is another aspect that motivated this decision, as the exchange of messages between the controller and the HSU is faster when they are on the same device.

Using an SDN controller in the scope of a residential environment could bring several advantages such as greater agility, more programmability, centralized data control, simplified operations, and better management of network resources. The HSU, for example, can send a blocking rule to the controller when it identifies a threat. In this way, the HSU takes advantage of the dynamism provided by the use of the controller to actively act on the network to mitigate threats that could harm users’ privacy.

Despite its benefits, adding an SDN controller in a residential scenario might not be a simple task for a user. However, for the scenario involving smart homes, a small device with low cost and good computing power can meet this need. The ISP or SSP can also provide an SDN-based device when offering such a solution for home users. The potential complexity for end users in dealing with an SDN controller is eliminated by adopting the FamilyGuard architecture, which performs all the management (communication) of the controller and provides APIs for developing web pages or mobile applications to control their devices and information.

### 4.3. Network Flow Generator (NFG)

The NGF component collects traffic that traverses a given network, or network segment, to generate [43] network flows. According to RFC 7011, a flow can be defined as a set of IP packets passing an observation point in the network during a specific time interval. Network flows have been used in several network applications ranging from troubleshooting connectivity issues to planning future bandwidth allocation. Here, we use network flows to identify and mitigate security issues.

Monitoring network flows can provide insights into how a network operates, its overall utilization, application usage, potential bottlenecks, anomalies that can signal security threats, etc. Several different standards and formats are used in monitoring network flows, including NetFlow [44], sFlow [45], and Internet Protocol Flow Information Export (IPFIX) [46].

The adoption of a solution to generate network flows is encouraged by the benefits of detecting anomalous traffic and other threats to network security. The information in the IP packet header provides the basis for generating network flows. The amount of data processed by the flow-based intrusion detection system is less, as it contains summary information. Another factor contributing to the decision to use network flows for analysis is the number of network applications that use end-to-end encryption. Since flow-based inspection only works with statistical features extracted from the packet header, this approach raises fewer privacy concerns than packet-based inspection because user information is protected from any intermediate scans.

Despite the benefits, flow-based intrusion detection also has some limitations. For example, the network flows represent a snapshot of summarized network traffic at a specific time. Therefore, it might be more difficult to distinguish some attack types [47].

### 4.4. Central Security Assistant (CSA)

The CSA aims to provide services that collaborate with the performance of the activities by the HSUs. The CSA can be provided by *Internet Service Providers* (ISPs) or by service providers interested in providing an adequate structure for the management of security in residential environments.

The flexibility in CSA positioning allows the architecture to be employed in the smart home context and in different IoT environments such as smart grid, healthcare, and others. However, it is necessary to evaluate, among other things, the number of devices deployed on the network and the traffic generated by them to define the hardware resources needed to meet the environment. One of the benefits of installing CSA on an ISP or cloud is the ability to sell security services to customers as models for detecting threats.

**Figure 5:**
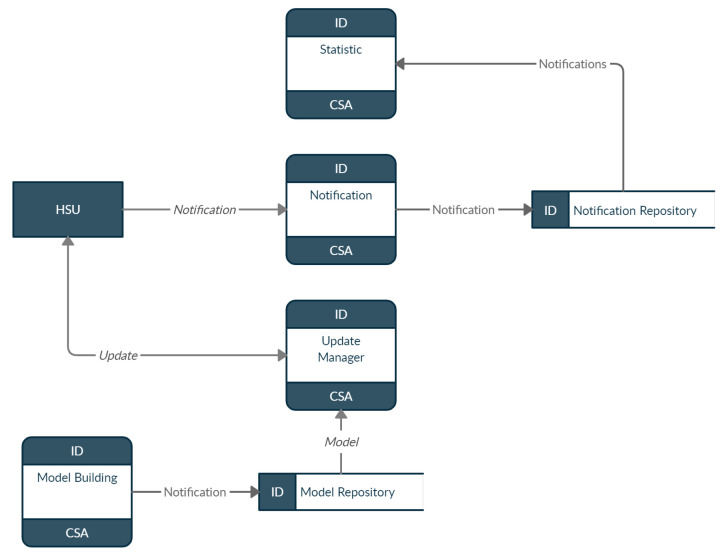
Communication between CSA components.

Figure 5 presents the data flow performed by the CSA, in which the notification process receives notifications provided by the HSU and saves them as notifications in the notification store. The CSA also runs the statistics process, which is responsible for creating metrics based on notifications received by the HSU. In addition to notifications, the CSA maintains the anomaly detection models used by HSUs.

Thus, the model building process is responsible for building these models and saving them in the repository. The update manager process can perform queries to check the models for updates and send messages notifying the HSU if there are any updates.

The architecture’s conceptual design aims to detect anomalies, allowing for the management of heterogeneous IoT devices deployed in smart home environments, and also focuses on the analysis of activities generated in the network. Therefore, a multi-layer structure was adopted that allows for the independent development of the components. Section 4.5 describes the logical layers of the FamilyGuard architecture.

### 4.5. FamilyGuard Layers

**Figure 6:**
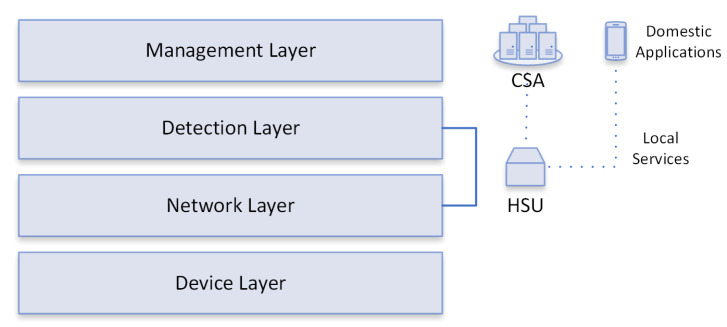
FamilyGuard layers and their relationship with HSU and CSA.

FamilyGuard is organized into four layers: Device layer, Network layer, Detection layer, and Management layer, depicted in Figure 6. The HSU and SDN controller operate at the Network and Detection layers. The Management layer hosts (i) the CSA, which helps different HSUs to perform their tasks, and (ii) applications, which help to control and configure local services that exist in residential environments. The structures of each layer are described below:

- Device Layer: represents all devices that can communicate in home environments, including laptops, smartphones, and smart devices such as sensors (temperature and presence) and actuators (light switches). There are several smart devices on the market, created by different manufacturers; therefore, residential environments are heterogeneous and complex for risk and threat management.
- Network Layer: has the ability to handle multiple protocols and receive/transmit data through the Devices layer, so that data packets are transferred over the data link, such as Wi-Fi, Ethernet, Wireless Sensor Network (WSN), and Machine- to-Machine (M2M).
- Detection Layer: performs anomaly detection (primary function) through well-defined services, from network traffic reception to notification, for layer management, by classifying a given flow as anomalous.
- Management Layer: is responsible for monitoring and controlling the settings of the residential environment through CSA and home control apps; the CSA collaborates so that HSUs can perform their functions through services that are essential for the functioning of the environment.

## 5. Validation

The validation consists of assessing some of the FamilyGuard functionalities described in Section 4. The idea here is to evaluate how FamilyGuard can cope with the major issues identified in the state-of-the-art solutions (Section 3): (i) network monitoring, (ii) machine learning models to mitigate threats, and (iii) deployment of the solution. With this in mind, we conducted some experiments using a version of FamilyGuard implemented in Python programming language and available at https://github.com/pedrodamaso/FamilyGuard.git (accessed on 1 February 2022), and also an exploratory investigation about the impact of FamilyGuard in some critical smart-home security operations. We empirically evaluated three aspects of FamilyGuard:

1. Implementation of HSU functionalities in a low-cost hardware—here, we want to answer some questions such as: is our architecture easy to adopt for heterogeneous residential environments? Is the deployment cost low? What hardware is needed to deploy the architecture components?
2. The ability of machine learning models to detect potential threats in HAN—the following questions guide this step: Is it possible to use unsupervised models to detect threats in the home environment? Does the traffic mixture between IoT and Non-IoT devices add additional complexity to the models? Do the benefits achieved with the models to provide additional protection mechanisms for the residential environment justify the adoption of the architecture?
3. HSU performance during the anomaly detection process—here, we consider some questions such as: How long, on average, does the HSU take to process a network flow and issue a decision on it? Is this time reasonable for decision making?

We also discuss the impact of FamilyGuard in dealing with the following situations:

4. We analyzed the risk of machine learning models becoming outdated and not providing an efficient threat detection rate because of changes in the environment, such as the addition of new devices and changes in network traffic behavior over time. Thus, we highlight some essential questions to mitigate this risk: How does one add or update the models used by the architecture? Who will provide these models? How will the model be made available?
5. We explore existing threat points in the residential environment that could impact the functioning of the FamilyGuard architecture.

Next, we will detail each of the aspects analyzed in FamilyGuard.

### 5.1. Implementation of HSU Functionalities in a Low-Cost Hardware

**Figure 7:**
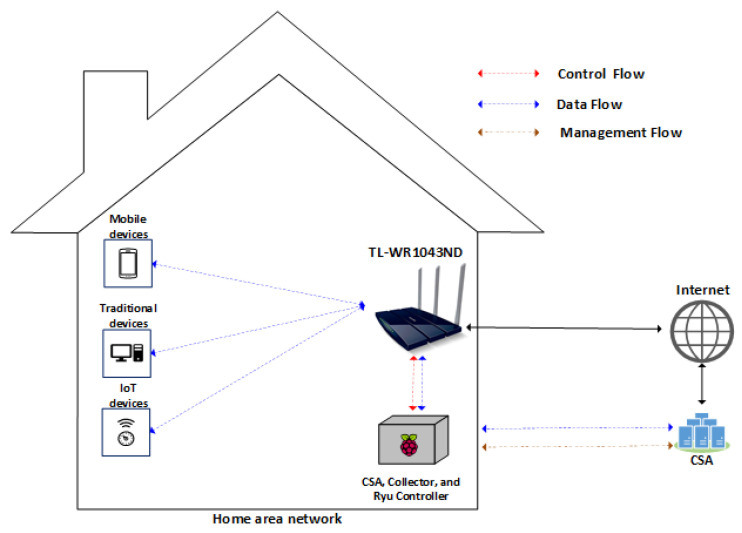
Experimental deployment of a home area network. The HSU is depicted as a junction of a TL-WR1043ND router and a Ryu controller.

The components described in Section 4.1, Section 4.2, Section 4.3 and Section 4.4 were deployed on a Raspberry Pi3 Model B Quadcore 1.2ghz, with 1GB of RAM and a 32 Gigabyte memory card, running on the Raspberry Pi OS. To deploy SDN on HAN, we used a TP-LINK TL-WR1043ND v3 router, with OpenWrt 18.06 and Open vSwitch 2.8.2 plug-ins. That way, when the HSU identified anomalies, it could proactively act, sending messages to the controller, to block the infected device. Figure 7 shows our deployment of FamilyGuard.

With this kind of deployment, our architecture can be used, at low cost, in several home environments. However, before determining whether the architecture is capable of providing additional protection for the home environment, we first need to evaluate the performance of the components installed on the Raspberry Pi. To do this, we monitored the memory and processor using RPi-Monitor tools while analyzing 78,836 network traffic flows to classify them into anomaly/non-anomaly flows using the OSVM classifier.

**Figure 8:**
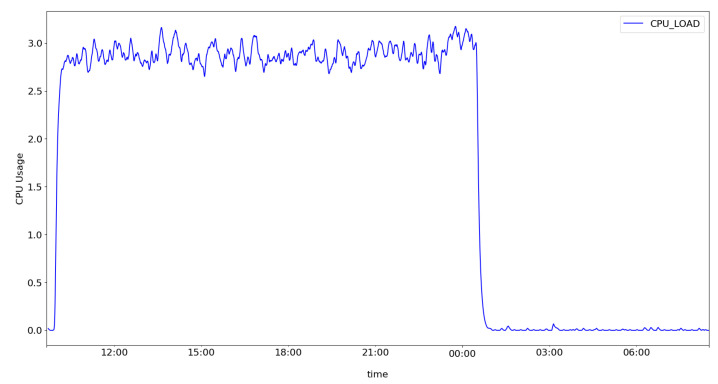
CPU performance during analysis of network flows.

**Figure 9:**
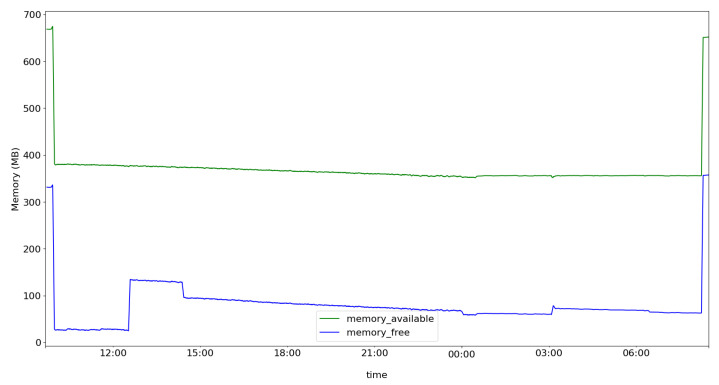
Memory and swap usage during analysis of network flows.

Figure 8 shows the average CPU load from the beginning of the execution of the architecture components to its completion. Figure 9 shows the amount of available memory, free memory, and swap. It is possible to notice that the CPU usage remains constant throughout the execution. Regarding the available memory, it is possible to observe that the y-axis of Figure 9 initially had about 690 MB of available memory. However, when bootstrapping the HSU module and loading the machine-learning models, the memory decreased to around 300 MB and remained constant until the end of the experiment.

For the analyzed example, the Raspberry Pi3 Pi 3 Model B supported the tests; however, if it were necessary to load new models, it is likely that their performance would not be satisfactory. Therefore, we recommend using Raspberry Pi 4 Model B with 4GB RAM as a minimum requirement to deploy the FamilyGuard architecture.

This experiment evaluated the performance of FamilyGuard components deployed on a Raspberry Pi3 Pi 3 Model B. In this way, we show that the architecture has a low implementation cost, minimal hardware configurations for the deployment of the architecture in the residential environment, and ease of use. However, we need to validate whether machine learning models can detect threats present in the environment. Section 5.2 details the analyzed algorithms.

### 5.2. Using Machine Learning to Detect Smart-Home Anomalies

The first step is to acquire network traffic information to portray the home environment. Data comprises traffic from IoT smart home devices and personal computers (PCs), such as mobile devices and laptops (non-IoT). The second step is to constitute traffic streams from packet capture files (PCAP), which are data files created using a program with packet data from a network. Third, network flows are decomposed into test cases to equate them with smart home environment scenarios. The last step corresponds to building the traffic classification model using unsupervised learning. We used three classic unsupervised learning algorithms (OCSVM, LOF, and IF) that have been applied in several anomaly detection applications [48,49]. After gathering all the PCAP files containing the captured network packets, the CICFlowmeter [50,51] tool was used to generate bidirectional flows.

Sivanathan et al. [52] suggested an environment with twenty-eight mechanisms containing cameras, lights, plugs, motion sensors, devices, and health monitors. They provided the PCAP files among the released data, including the raw network packets. Available traffic spans both IoT and non-IoT device communications. Therefore, we remove all non-IoT devices to match the IoT dataset. The withdrawn devices included traffic from an HP printer, Android smartphone, laptops, iPhone, Samsung Galaxy Tab, and a PIX-STAR Photo-frame.

To portray traffic from non-IoT devices, we chose the dataset provided by the Canadian Institute for Cybersecurity (CIC) [50], encompassing the different types of traffic and applications known by the dataset: browsing, email, chat, streaming, file transfer, VoIP, and P2P.

For the clustering of non-IoT data, samples of distributed denial-of-service (DDoS) attacks provided by [53] were used. We introduced the following types of attacks into our dataset: Distributed Reflection-Based Denial of Service (DDoS) using NETBIOS, SYN Flood, and UDP Flood. In summary, for anomalous IoT traffic, we used data provided by the Stratosphere Research Laboratory (SRL) [54] and selected network traffic related to CoinMiner, Muhstik, and Mirai botnets.

We created three datasets, or test cases (TCs), to evaluate our experiments. The first one, TC1, mixes IoT and non-IoT device traffic. The second, TC2, is derived from TC1, containing only the examples referring to non-IoT devices traffic. Finally, in the third one, TC3, only the IoT device samples were taken from the first test case. Our goals with these experiments include: evaluating the feasibility of using unsupervised learning algorithms to detect anomalies in the residential environment; verifying whether the mixture of IoT and Non-IoT network flows might harm the anomaly detection process; and assessing the HSU performance during the anomaly detection process (experiments described in Section 5.3). Table 2 summarizes the test cases, the type of traffic that constitutes each one, and the number of flows used in the training and testing phase.

We selected the following metrics to evaluate the performance of models:

- True Positive Rate (TPR), sensitivity or recall is the part of positive examples that the model correctly predicted.
- True Negative Rate (TNR) is the portion of negative test examples that the model predicts without errors.
- Error Rate (ER) is the part of the test suite examples that the model erroneously predicts.
- Precision is related to the test examples that the model correctly predicts.
- Computing Field Under AUC corresponds to how much the model can differentiate between classes. The more considerable the measure, the better the model predicts negative classes like anomalies and positive classes like regular traffic.

This subsection presents the evaluation of three classifiers in three test cases depicting different home network scenarios. Our objective is to assess whether OCC algorithms are enabled to provide additional protection for the residential environment by detecting anomalies using network traffic flows. Table 3 summarizes our results. The Ref field. alludes to the reference of the related algorithm.

The results presented in Table 3 suggest that some algorithms may perform better than others, but statistical tests need to be conducted to confirm this. We also conclude that it is possible to use unsupervised models to detect threats in the residential environment, and the combination of traffic from IoT and non-IoT devices adds complexity to identifying anomalous behaviors. Through the experiments, we highlight that the models can provide additional protection mechanisms that justify the adoption of the architecture.

### 5.3. Hsu Performance during the Anomaly Detection Process

To assess whether the models can identify a threat in a viable time so that it is possible to take some preventive action, we analyzed three test cases. We monitored how long the models took to make their predictions. That is, the time it takes HSU to analyze the network traffic flow.

**Figure 10:**
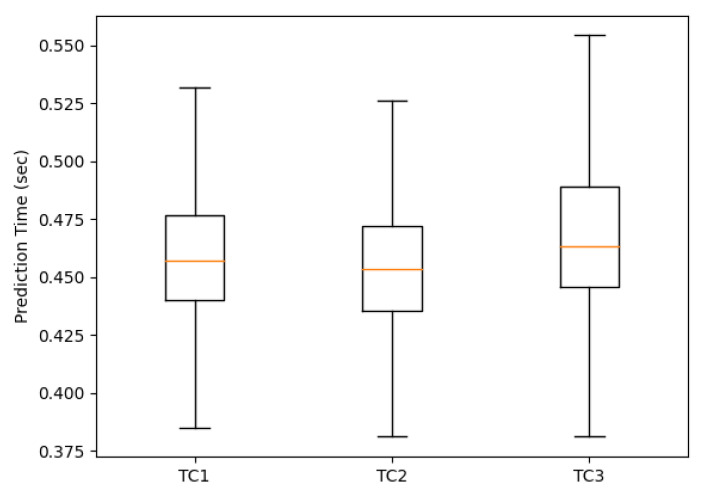
Average prediction time on test cases TC1, TC2, and TC3.

Figure 10 shows the average time taken, from flow arrival to prediction. In TC1, 78.836 flows were analyzed, in TC2, 39.495 flows were analyzed, and in TC3, 39.341 flows were analyzed. Details about each of the test cases are presented in Section 5.2.

It is possible to notice that the average time for the prediction is between 0.37 and 0.55 s by network flow. Considering that we use low-cost equipment, this can be seen as a relatively short time.

We consider that the time obtained satisfies the needs of home users; for example, upon receiving a DDoS attack to make some service unavailable in the environment, our architecture is able to identify and react proactively by sending a rule to the SDN controller to block the threat in a relatively short time.

Therefore, we evaluated the ability of the Raspberry Pi3 Pi 3 Model B to support the architectural components and the response time that the models take to perform a prediction. In that way, we conclude that the time spent is enough to make decisions to mitigate the identified threats. However, another factor of great importance is providing a residential environment with a better level of security and privacy, which means the ability to deal with changes in each domain. An example of this is adding a known device to the network. In this way, the validation in Section 5.4 seeks to describe the solution proposed by our architecture.

### 5.4. Process of Updating Machine Learning Models in HSU

To deal with the inclusion of new devices and the placing of new protocols in heterogeneous residential environments, the HSU relies on the help of the CSA specified in Section 4.4, which aims to assist a group of HSUs in the detection of anomalies.

To determine if a flow is anomalous, the models used by the HSU are not trained or updated based on the residential traffic that they control, so there is no risk of learning from malicious traffic existing in the monitored environment.

Another reason for outsourcing the creation of detection models is that they demand a lot of computational power, making it impossible to use a low-cost device, such as the Raspberry Pi3. In this way, we transfer the responsibility for training the model to SPPs. SSPs can provide the CSA, which has the role of creating the machine learning models and making them available on the HSU with anomalous traffic. The creation of models is under the responsibility of specialized security service providers, as the definition must be free from anomalies.

Suppose a residential environment has an anomaly detection model for IoT devices. This environment has three devices (e.g., Amazon Echo, Netatmo Welcome, and Belkin Wemo motion sensor); however, based on the needs of the residents of the residential environment, a new IoT device needs to be added, for example, a smart baby monitor. The detection model needs to be updated to prevent this new traffic from being classified as an anomaly.

The user (resident) can contact the service provider and request a suitable model, including all the devices he has in his residence. The service providers can offer models based on the identification of residential traffic, making this process automatic. For example, when identifying a new device on the network, the service provider already makes an updated model for the environment. In this way, we present how the models are updated and made available to the architecture and how the CSA contributes to this process.

### 5.5. Threats Targeting the HSU and Devices Present in the Residential Environment

**Figure 11:**
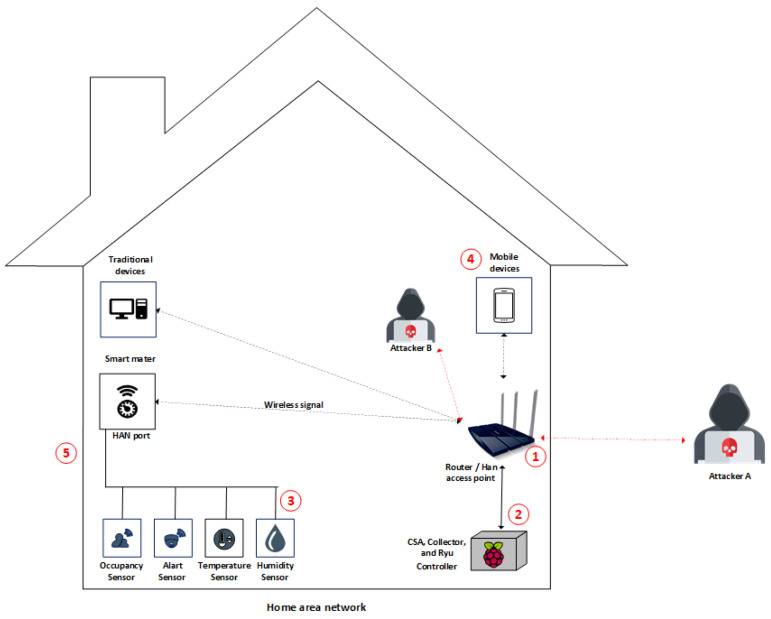
Threat points that can compromise the security of home area network.

The FamilyGuard architecture provides additional security mechanisms for residential environments; thus, it is necessary to analyze threats that intruders can exploit. Based on [59,60], we define five threat points. Figure 11 illustrates these points. At threat point 1, “Attacker A” can exploit the following threats:

- Eavesdropping: monitoring network traffic without the authorized users knowing about it. Communication may contain sensitive data that home users do not want to be discovered by unauthorized users.
- Masquerading: an attacker can acquire certain unauthorized advantages by justifying being an alternate legitimate user (e.g., guest). The attacker can impersonate an unauthorized home user and remotely access the smart home’s internal network system, considering that the ultimate goal is to gather confidential data or acquire services.
- Replay attack: an attacker first receives messages that are legitimately exchanged between two parties and then re-transmits them as an authorized party.
- Message modification: can occur when attackers intend to hijack communications between two factual parties, for example, by modifying the software to make it act maliciously or changing values in the data.
- Denial of Service: an attacker attempts to attack the availability of the network. The attacker may send very large messages, or message bursts, to the smart home networking system with the intention of overloading its services. In this way, genuine users cannot obtain the services from the home network.

At threat point 2, “Attacker B” has already gained authorized access to the environment and seeks to take down the additional protection service that identifies anomalous flows; thus, the intruder can send a huge amount of messages to devices running the HSU and controller connected to the Internet to restrict internal traffic transmitted by wired or wireless networks inside the smart home.

Threat point 3, “Attacker B”, can deplete IoT device resources, making their use unfeasible. To mitigate such threats, we use anomaly detection based on network traffic flows. Therefore, experiments and analyses are presented in Section 5.2, which show the possibility of dealing with possible threats with the support of artificial intelligence.

Finally, at point 4, compromised external devices can join the home network, causing damage and exploiting flaws in the internal environment and, at point 5, an attacker can listen to traffic outside the home to capture sensitive data or infer which internal devices are present in the home or if there is communication at any given time.

## 6. Limitations

Although Section 5 showed the validation of several functionalities and discussed how the proposed architecture advances the state of the art, it is important to highlight some limitations and threats to validity.

In the first validation described in Section 5.1, we showed thethe simplicity of the architecture by deploying it in low-cost hardware. We monitored the behavior of a Raspberry Pi3 Model B during the analysis of 78,836 network traffic flows. Although we demonstrated that using the architecture components with a low-cost device is possible, we cannot say that this behavior will be the same as other models available on the market.

In the second validation presented in Section 5.2, we conducted experiments using machine learning models to detect threats in the residential environment. In this experiment, we used unsupervised models to detect threats. However, the number of threats in the experiments is too small to determine if the model is still valid in a scenario with multiple threats; further experiments and detailed study are required. Likewise, we can say that separating IoT traffic from Non-IoT facilitates the identification of threats in the experiments performed. Still, the number of threats and amount of data used in the study does not guarantee the same behavior in a more complex scenario. The most critical point of this validation was to use a model to detect threats in real time in the residential environment to validate the communication between the architecture components. A severe limitation found during this experimental validation was the lack of public datasets containing both normal and attack samples from residential traffic. We circumvented this issue by creating our own dataset. It is important to evaluate FamilyGuard architecture using other datasets.

The fourth validation described in Section 5.4 explores the foci of threats that are harmful to the residential environment, that is, that try to harm the privacy of people living in such an environment. It is possible to mitigate threats for the points described; however, due to the dynamic environment and constant changes concerning the types of devices and protocols, new threats arise over time. In this scenario, we also have the human factor and the lack of technical knowledge about information security. Another factor contributing to new threats is the lack of software and hardware updates. Based on all the threats cited, security and availability of the architecture are concerns that will require detailed research.

Finally, as mentioned in the last validation presented in Section 5.5, the objective of this experiment was to demonstrate how machine learning models are updated to handle changes in the behavior of home network traffic caused by the addition of new devices and protocols. Updating the models used by architecture is simple. However, the main concern is about the quality of the models and whether they will be effective for the residential environment. It was not presented in this article, but the components proposed in the CSU and HSA architecture allow for the implementation of collaborative threat detection models, a solution that can improve the quality of the models available to the residential environments.

## 7. Conclusions and Future Work

This work proposes FamilyGuard, a security architecture designed to manage residential environments efficiently and effectively by using low-cost hardware. We described and evaluated a complete scenario for anomaly detection. Our results indicate that: (i) it is possible to deploy the architecture using a Raspberry Pi3 Model B; (ii) machine learning models can provide additional protection to the residential environment; and (iii) machine learning models installed on low-cost hardware can make predictions in a viable time frame to mitigate identified threats. We also discussed how the proposed architecture deals with the model update task and some of the threats that may exist in residential environments. We contribute to the scientific community by presenting the design, implementation, testing, and validation of a machine-learning-based security architecture for smart homes.

Future work will be directed to evaluating the performance of one-class algorithms for several types of security threats and to identifying the best algorithms according to different smart home scenarios. We also intend to explore the idea of building collaborative models to improve threat detection. Although this work presents models for anomaly detection based on network flows, this was just an experiment to validate the components and the use of the architecture in a real environment. However, it is possible to explore several possibilities that can improve the safety and comfort of people living in a given environment, for example, models to optimize energy consumption, detect occupancy within a certain room of the house to check the perimeter for intruders, recognize abnormal activity or events to detect dangerous situations such as fire or flood, or monitor activities of the elderly.

## Figures and Tables

**Table 1:** Characteristics of architectures.

Name	Ref	Year	Placement Strategy	Validation Strategy	Possible Threats	Security Goals Compromised	Countermeasures	Techniques/Tools	SDN
FamilyGuard		2022	Edge/Cloud	Empirical Experimental	DDoS Replay attack Eavesdropping	Availability Integrity Confidentiality	Anomaly detection	Unsupervised algorithms LOF, OCSVM, IF	•
CBB	[10]	2021	Edge/Cloud	Experimental	Message Modification Replay attack Eavesdropping	Availability Integrity Authenticity Confidentiality	Blockchain-based	Chaincode	∘
Urban Patrol	[11]	2021	Edge/Cloud	Experimental	Sinkhole Attack Worm Attack Side Channel Attack	Availability Confidentiality Integrity Authenticity	Anomaly detection Blockchain RBA	ML XGBoost	∘
TSP2	[37]	2020	Edge	Theoretical	Replay Attacks Data Leakage Eavesdropping	Confidentiality	Cryptography	Lightweight cryptography algorithms	∘
EGRBAC	[12]	2020	Edge/Cloud	Experimental	Man-in-the-Middle Identity misbinding	Authenticity	RBAC	Custom model	∘
GHOST	[7]	2019	Edge/Cloud	Theoretical	Impersonation Replay attack	Integrity Availability	Anomaly detection Blockchain	-	•
SHSec	[8]	2019	Edge	Experimental	DDoS	Availability Confidentiality	Anomaly detection	Conditional probability distribution	•
HNR	[9]	2018	Edge/Cloud	Experimental	DDoS	Availability	Autonomous management	Fault detection	•
Securebox	[39]	2017	Edge/Cloud	Experimental	DDoS	Integrity Availability	IPS	SNORT	•
HanGuard	[40]	2017	Edge	Empirical	Man-in-the-Middle Identity misbinding	Authenticity	Control policies	Custom model	∘
CG	[41]	2017	Edge/Cloud	Experimental	DDoS	Availability	IPS	SNORT	∘

**Table 2:** Evaluation of the classification models installed on the HSU.

Test Case	Traffic	Type	Training	Training Oversampling	Test
TC 1	Non-IoT	Normal	161,579	272,539	35,907
	NetBIOS	Anomaly	-	-	1196
	SYN	Anomaly	-	-	1196
	UDP	Anomaly	-	-	1196
	IoT	Normal	290,347	272,539	35,764
	Muhstik	Anomaly	-	-	1193
	Mirai	Anomaly	-	-	1192
	Coinminer	Anomaly	-	-	1192
TC 2	Non-IoT	Normal	161,579	272,539	35,907
	NetBIOS	Anomaly	-	-	1196
	SYN	Anomaly	-	-	1196
	UDP	Anomaly	-	-	1196
TC 3	IoT	Normal	290,347	272,539	35,764
	Muhstik	Anomaly	-	-	1193
	Mirai	Anomaly	-	-	1192
	Coinminer	Anomaly	-	-	1192

**Table 3:** Summary of results.

Classifier	Refs	Test Case	TPR	TNR	ER	ACC	AUC
OCSVM	[55]	TC1	0.4527	0.9007	0.1399	0.8600	0.6767
		TC2	0.8667	0.8985	0.1043	0.8956	0.8826
		TC3	0.3958	0.8993	0.1464	0.8535	0.6476
LOF	[56]	TC1	0.9157	0.8684	0.1272	0.8727	0.8920
		TC2	0.9693	0.8561	0.1335	0.8664	0.9127
		TC3	0.8660	0.8980	0.1048	0.8951	0.8820
IF	[57,58]	TC1	0.4654	0.9004	0.1391	0.8608	0.6829
		TC2	0.9200	0.9005	0.0976	0.9023	0.9102
		TC3	0.3936	0.8979	0.1478	0.8521	0.6457
